# Rapid cycling genomic selection in maize landraces

**DOI:** 10.1007/s00122-025-04855-6

**Published:** 2025-03-17

**Authors:** Clara Polzer, Hans-Jürgen Auinger, Michelle Terán-Pineda, Armin C. Hölker, Manfred Mayer, Thomas Presterl, Carolina Rivera-Poulsen, Sofia da Silva, Milena Ouzunova, Albrecht E. Melchinger, Chris-Carolin Schön

**Affiliations:** 1 Plant Breeding, TUM School of Life Sciences, Technical University of Munich, 85354 Freising, Germany; 2 Present Address: KWS SAAT SE & Co. KGaA, 37574 Einbeck, Germany; 3 Institute of Plant Breeding, Seed Science and Population Genetics, University of Hohenheim, 70593 Stuttgart, Germany; 4 Present Address: Bayer Crop Science, 46325 Borken, Germany

## Abstract

**Key message:**

**A replicated experiment on genomic selection in a maize landrace provides valuable insights on the design of rapid cycling recurrent pre-breeding schemes and the factors contributing to their success.**

**Abstract:**

The genetic diversity of landraces is currently underutilized for elite germplasm improvement. In this study, we investigated the potential of rapid cycling genomic selection for pre-breeding of a maize (*Zea mays* L.) landrace population in replicated experiments. We trained the prediction model on a dataset (N = 899) composed of three landrace-derived doubled-haploid (DH) populations characterized for agronomic traits in 11 environments across Europe. All DH lines were genotyped with a 600 k SNP array. In two replications, three cycles of genomic selection and recombination were performed for line per se performance of early plant development, a major sustainability factor in maize production. From each cycle and replication, 100 DH lines were extracted. To evaluate selection response, the DH lines of all cycles and both replications (N = 688) were evaluated for per se performance of selected and unselected traits in seven environments. Selection was highly successful with an increase of about two standard deviations for traits under directional selection. Realized selection response was highest in the first cycle and diminished in following cycles. Selection gains predicted from genomic breeding values were only partially corroborated by realized gains estimated from adjusted means. Prediction accuracies declined sharply across cycles, but only for traits under directional selection. Retraining the prediction model with data from previous cycles improved prediction accuracies in cycles 2 and 3. Replications differed in selection response and particularly in accuracies. The experiment gives valuable insights with respect to the design of rapid cycling genomic selection schemes and the factors contributing to their success.

**Supplementary Information:**

The online version contains supplementary material available at 10.1007/s00122-025-04855-6.

## Introduction

A major goal of plant breeding research is to devise breeding schemes that maximize selection gain. While elite breeding programs are revised constantly to accommodate technological advances such as genome-based prediction, new technologies are still underutilized in programs harnessing genetic variation from landraces. High hopes lie in the use of landraces for broadening the genetic basis of elite germplasm and sustaining long-term selection gain, but a large performance gap with elite material and their heterogeneous and heterozygous nature have hampered broad applicability (Sood et al. 2014). A major challenge in utilizing landraces for quantitative trait improvement is to select for favorable alleles of target traits not present in elite germplasm and at the same time disentangle them from undesired alleles by recombination. To meet these goals, multi-trait genomic selection can be applied, but practical examples are sparse and optimization of genomic pre-breeding has mostly been studied in simulations (Gorjanc et al. 2016; Sanchez et al. 2023).

Gorjanc et al. (2016) compared different pre-breeding strategies for maize (*Zea mays* L.) landraces in silico. They concluded that for polygenic traits, landrace-derived material should not be introgressed into elite backgrounds too early, because subsequent selection favored elite over landrace haplotypes. Instead, they recommended to apply recurrent genomic selection to increase the frequency of favorable alleles in the respective populations before crossing with elite germplasm. Hölker et al. (2022) presented theoretical and experimental results on genome-based prediction in landrace-derived maize populations. They estimated prediction accuracies for several agronomic traits in doubled-haploid (DH) lines derived directly from individual landrace S_0_ plants and in S_1:2_ lines derived from crosses of landrace S_0_ plants with an inbred line. Prediction in DH lines was superior to the alternative approach for all traits. Prediction accuracies were as high as 0.61 for early plant height in DH lines per se and 0.58 for biomass yield in their testcrosses. These results suggest that application of genomic selection can open new avenues for the improvement of quantitative traits in landrace-derived populations.

When evaluating the efficiency of recurrent selection programs, selection gain is generally determined per cycle or year. Thus, the length of the breeding cycle, i.e., the time between recombinations, is of utmost importance (Cobb et al. 2019). In phenotypic selection of outcrossing species, cycle length is shortest for mass selection of heterozygous S_0_ plants. For traits that can be evaluated on individual plants like resistance traits or distinctive plant and ear morphology traits, mass selection can be effective, but for most quantitative traits, phenotypic mass selection is not an option, because S_0_ plants cannot be replicated in space and time and phenotypes are the result of additive and dominant gene action (Hallauer 1992). In contrast, mass selection on genomic breeding values does not suffer from these limitations. Genomic selection is independent of the environment and, depending on the model used for training, can act on additive as well as dominance genetic effects. Given a high-quality dataset for training the prediction model, genome-based prediction should allow selection for complex traits on heterozygous plants. In this context, the most crucial question is, how much selection response can be expected from genomic selection in multiple consecutive cycles without time-consuming retraining. Experimental results on the efficacy of this type of population improvement are sparse and there are still many unknowns on how to design such fast-track selection schemes based on genomic breeding values.

This study aimed at evaluating rapid cycling genomic pre-breeding of maize landraces experimentally. The main goal was to accelerate plant development in the juvenile stage but simultaneously avoid changes in final plant height to minimize effects on lodging. Fast development of maize roots and shoots will improve resource efficiency of the crop with respect to nutrients and water available in early spring. Faster soil coverage will preserve soil fertility and reduce the requirement for herbicide treatment. Reduced cold sensitivity in the juvenile stage will allow earlier planting and increase biomass production by extending the vegetation period. In addition, the risk of potential summer drought will be mitigated through a more rapid development during the vegetative phase and earlier onset of flowering. While genetic variation for early plant development is limited in elite flint germplasm (Strigens et al. 2013), we could show that flint maize landraces carry unique beneficial haplotypes for early vigor and early plant height not found in a broad sample of elite founder lines (Mayer et al. 2020; Urzinger et al. 2025). To harness the native diversity inherent in these resources, we initiated a selection experiment from a DH library described by Hölker et al. (2019). A genomic best linear unbiased prediction model was trained on these data and the best lines derived from the landrace Petkuser Ferdinand Rot were selected and recombined based on a multi-trait criterion in three selection cycles with two replications of the same experiment. Selection progress was monitored at the homozygous level with DH lines extracted from each selection cycle within each replication together with a random sample of the original population in multi-environment trials.

We addressed the following research questions: (i) What is the realized selection response for line per se performance over cycles? (ii) How do estimates of quantitative genetic parameters and prediction accuracy vary across DH populations derived from each cycle of selection? (iii) Are there significant differences between the two replications of the selection scheme?

## Materials and methods

### The training set

Model training for all selection steps was based on a data set (PKL) comprising 899 flint doubled-haploid (DH) lines derived from the landraces Petkuser Ferdinand Rot (PE, Germany, N = 402), Kemater Landmais Gelb (KE, Austria, N = 471), and Lalin (LL, Spain, N = 26) (Mayer et al. 2022). Phenotypic data collection and analysis are described in detail in Hölker et al. (2019). Briefly, DH lines were evaluated for per se performance of 25 traits in replicated trials in up to 11 environments across Europe during 2017 and 2018. In our study, the following traits were used: early plant height at growth stages V4 and V6 (PH_V4 and PH_V6, measured as length of the longest, manually upright stretched leaf at the corresponding stage, averaged over three plants per plot, cm); final plant height (PH, cm), and female flowering (FF, days to silking after sowing). The DH library was genotyped with the 600 k single nucleotide polymorphism (SNP) Affymetrix^®^ Axiom^®^ Maize Array (Unterseer et al. 2014).

Genomic estimated breeding values (GEBVs) were obtained by genomic best linear unbiased prediction (GBLUP) with the model

1 $$\varvec{y = X\mu + Zu + e,}$$

where ${\varvec{y}}$ is a vector of adjusted entry means of the 899 (*n*) DH lines from data set PKL. ${\varvec{\mu}}$ refers to the vector of fixed effects for the landraces, ${\mu }_{j}$, with *j* = 1, 2, 3 denoting PE, KE, and LL, respectively. ${\varvec{u}}$ is a *n*-dimensional vector of the random genotypic effects of the DH lines with ${\varvec{u}}\boldsymbol{ }\sim N(0,\mathbf{K}{\sigma }_{g}^{2})$, where ${\sigma }_{g}^{2}$ is the genetic variance. $\mathbf{K}$ denotes a (*n* x *n*) realized genomic relationship matrix calculated according to method 1 of VanRaden (2008). ${\varvec{e}}$ is a *n*-dimensional vector of random residuals, with ${\varvec{e}} \sim N(0,\mathbf{I}{\sigma }^{2})$, where $\mathbf{I}$ is an (*n* x *n*) identity matrix and ${\sigma }^{2}$ is the error variance. ${\varvec{X}}$ and ${\varvec{Z}}$ are incidence matrices, linking ${\varvec{\mu}}$ and ${\varvec{u}}$ with ${\varvec{y}}$. GEBVs per DH line and trait were calculated using the R package ASReml-R (Butler et al. 2009).

### Selection and recombination

The aim of the selection experiment was to evaluate the realized selection response for three traits over three cycles of selection, with two cycles on heterozygous individuals without phenotypes. For early plant development in stages V4 and V6, we performed directional selection, for final plant height we applied stabilizing selection to prevent an undesired correlated selection response. To this end, the three selection traits were combined in a multi-trait genomic selection criterion (SC) in all three cycles. First, GEBVs of the three traits were calculated by extending Eq. 1 to a multi-trait model. Second, GEBVs for each trait were mean centered and scaled by their respective standard deviations. GEBVs for final plant height were transformed (PH_trans) by subtracting the absolute value from the maximum value of the respective selection population. Third, for each genotype $i$ the selection criterion ${SC}_{i}$ was calculated as a linear combination of the multi-trait GEBVs for early and transformed final plant height:

2 $$SC_{i} = PH\_V4_{i} + PH\_V6_{i} + 2*PH\_trans_{i}$$

Genotypes were ranked according to the SC (Eq. 2). We restricted selection to one landrace (PE) to avoid the effects of population structure in subsequent analyses. Thus, the 20 highest-ranking DH lines were selected from the 402 DH lines derived from landrace PE (C0) and split by even and odd ranks, forming two sets of founder lines (C0sel) for the two replications (R1 and R2) of the selection program.

**Fig. 1:**
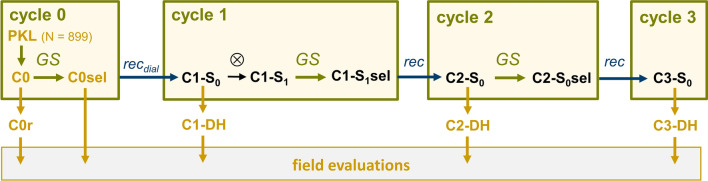
Rapid cycling genomic selection (GS) scheme for early and final plant height traits. Following model training with a set of 899 DH lines, the best C0sel DH lines from population PE (C0) were selected based on a multi-trait selection criterion, recombined in a diallel crossing scheme and selfed once to produce the C1-S_1_ population. Subsequently, two cycles of GS and mating of the selected candidates in pairs were conducted. From each selection cycle, DH lines were sampled (C0r) or produced (C1-DH, C2-DH, C3-DH) and evaluated jointly in field trials

In each replication, the same genomic selection scheme was followed (Fig. 1). The 10 C0sel founder lines were mated in a diallel crossing scheme and subsequently self-pollinated for production of ca. 1000 S_1_ plants (C1-S_1_). In R2, two selected lines failed to produce progeny, so that only eight DH lines served as founders of subsequently derived populations. C1-S_1_ plants were genotyped with 11 k SNPs (see below). Multi-trait GEBVs were calculated as described above (Eq. 1) with the realized genomic relationship between the training and the prediction set calculated from the 11 k markers. Approximately 40 C1-S_1_ plants (C1-S_1_sel) were selected and crossed in pairs to produce 20 full-sib families whereby parents were chosen based on high modified Rogers’ distance [MRD; (Wright 1978)] between them. From ca. 1000 resulting C2-S_0_ plants, genotypic data of 11 k SNPs were used for calculating multi-trait GEBVs (Eq. 1). About 30 were selected (C2-S_0_sel) and the offspring of 15 crossing pairs maximizing MRD represented the C3-S_0_ population. To maintain a high effective population size, we applied a restriction on the number of offspring per C0sel founder line. The exact numbers of genotypes available for each step in R1 and R2 of the selection program are given in Fig. S1. In addition, the size of the full-sib families and number of progenies selected from them are given in Table S1.

### DH population development and field evaluations

For evaluating the selection progress in each replication at the level of fully inbred materials, we generated approximately 100 DH lines from each selection cycle by in vivo haploid induction according to established procedures (Chaikam et al. 2019) (Fig. 1, Fig. S1). Further, we randomly drew a set of 105 DH lines (C0r) out of the 402 DH lines from the initial source population PE as a baseline for comparisons across cycles.

Line per se performance of 688 DH lines from the seven populations (C0r, C1-DH-R1, C2-DH-R1, C3-DH-R1, C1-DH-R2, C2-DH-R2, C3-DH-R2) was evaluated together with the C0sel founder lines, 11 inbred checks, and the PE landrace population. Field trials were conducted using alpha lattice designs with incomplete blocks of 10 plots at three locations (Oberer Lindenhof (OLI), Einbeck (EIN), Bernburg (BBG)) in 2022 and 2023 and in Roggenstein (ROG) in 2022, resulting in seven environments (location × year combinations) covering a wide range of agro-climatic conditions in Germany. C3-DH lines were evaluated in 2023 only. Twenty kernels were sown in single-row plots of 3 m length at a distance of 0.75 m, resulting in a plant density of ca. 9 plants per m^2^. Fertilization and plant protection were carried out according to standard agricultural practices. Phenotypic trait collection was the same as described for the training set (Table S2). Phenotypic observations from plots containing at least five uniform plants were filtered by a Grubbs’ outlier test (Grubbs 1969).

### Analyses of phenotypic data

The statistical model for analyses of the field trials described above was

3 $$y_{pikst} = {\upmu }_{p} + g_{i\left( p \right)} + l_{k} + gl_{ik\left( p \right)} + r_{s\left( k \right)} + b_{{t\left( {sk} \right)}} + e_{pikst}$$

where ${y}_{pikst}$ is the phenotypic plot level observation. ${\mu }_{p}$ refers to the fixed effect of population *p* with *p* = 1 to 7 for each of the seven DH populations, *p* = 8 for the 10 C0sel-R1 lines, *p* = 9 for the 8 C0sel-R2 lines, and *p* = 10 for checks. ${g}_{i(p)}$ is the effect of genotype *i* nested within population *p*. For variance component estimation, ${g}_{i(p)}$ was treated as fixed for *p* = 8 to 10 and as random for *p* = 1 to 7 with ${g}_{i(p)}\sim \text{ iid }N(0,{\sigma }_{g(p)}^{2})$, allowing for heterogeneous genetic variances among populations. For obtaining adjusted entry means per genotype, ${g}_{i(p)}$ was treated as fixed for *p* = 1 to 10. ${l}_{k}$ is the random effect of environment *k,* ${gl}_{ik(p)}$ the random interaction effect for genotype *i* with environment *k*, nested in population *p*, with heterogeneous variances ${\sigma }_{gl(p)}^{2}$ among populations, ${r}_{s(k)}$ and ${b}_{t(sk)}$ are random effects of replication *s* nested in environment *k* and incomplete block *t* nested in replication *s* in environment *k*. ${e}_{pikst}$ is the random residual error of observation *pikst*, with ${e}_{pikst}\boldsymbol{ }\sim \text{ iid }N(0,{\sigma }_{e}^{2})$ and ${\sigma }_{e}^{2}$ denoting the error variance. Adjusted entry means and variance components of random effects were estimated using the restricted maximum likelihood method implemented in the R package ASReml-R (Butler et al. 2009). Heritabilities on an entry-mean basis and their confidence intervals were calculated applying established procedures (Hallauer et al. 2010; Knapp et al. 1985).

### Genotypic data

In each replication, C1-S_1_, C2-S_0_, and the DH lines from cycles C1 to C3 were genotyped using a 15 k SNP custom Illumina^®^ Array, proprietary to KWS SAAT SE & Co. KGaA. In total, 93% of these SNP markers overlapped with the 600 k Affymetrix^®^ Axiom^®^ Maize Array (Unterseer et al. 2014), used for genotyping the training set.

Filtering steps for excluding genotypes and markers were performed separately for heterozygous plants and DH lines as described by Mayer et al. (2022). In addition, we validated pedigrees of S_1_ and S_0_ plants as well as DH lines by comparing their marker profiles with those of their parents. Missing marker calls were imputed with BEAGLE v5.4 (default parameters) (Browning et al. 2018). In total, 11,160 SNP markers, subsequently referred to as 11 k SNP set, common to all genotypes and overlapping with markers from the 600 k chip remained for all further analyses. A total of 3,915 S_0_ and S_1_ genotypes and 985 DH lines remained after quality check. Based on these genotypes, we performed a principal coordinate analysis (Gower 1966) on MRD calculated from allele frequencies of populations.

### Response to selection

Response to selection was evaluated based on two approaches: (i) GEBVs of all genotypes (DH lines or heterozygous S_0_ and S_1_ individuals), and (ii) adjusted entry means of the DH lines estimated from field trials in 2022/23. Comparisons were made between (i) the selection cycles within each replication, (ii) the two replications of the selection experiment, and (iii) heterozygous populations and their corresponding DH populations (GEBVs only).

Mean GEBVs were calculated for each of the 14 populations (C0, C0r, C0sel-R1, C0sel-R2, C1-S_1_-R1, C1-S_1_-R2, C2-S_0_-R1, C2-S_0_-R2, C1-DH-R1, C1-DH-R2, C2-DH-R1, C2-DH-R2, C3-DH-R1, C3-DH-R2). For the three selection traits, GEBVs were obtained from the multi-trait model, for flowering time from a single-trait model. Data set PKL (N = 899) was used for model training, but for calculating the respective realized genomic relationship matrices, allele frequencies were derived from landrace PE to set the mean GEBV of population C0 to zero (Table 1). The response to selection across DH populations is expressed in units of the standard deviation (SD) of GEBVs in C0.

**Table 1:** Mean GEBVs of DH, S_0_ or S_1_ populations for plant height in the V4, V6, and final growth stages (PH_V4, PH_V6, PH) and days to female flowering (FF) in cycles C0 to C3

	N		PH_V4		PH_V6		PH		FF	
	R1	R2	R1	R2	R1	R2	R1	R2	R1	R2
C0	402		0		0		0		0	
C0sel	10	8	5.29	6.35	8.64	10.59	6.30	10.91	0.47	− 1.23
C1-S_1_	1003	1001	5.25	6.39	8.60	10.67	6.14	10.90	0.57	− 0.95
C1-DH	100	100	5.66	5.97	8.99	9.65	7.03	10.25	− 0.35	− 1.38
C2-S_0_	1005	906	7.14	8.34	11.74	14.09	7.49	14.25	0.30	− 1.61
C2-DH	104	111	7.15	8.18	11.96	13.76	8.37	12.24	− 0.56	− 1.52
C3-DH	90	78	7.97	9.66	13.09	16.08	7.72	15.53	− 0.01	− 2.20
*Slope*										
C1-S_1_—C2-S_0_			1.9*	2.0*	3.1*	3.4*	1.4*	3.4*	− 0.3*	− 0.7*
C1-DH—C3-DH			1.2*	1.9*	2.1*	3.3*	0.4	2.6*	0.2	− 0.4*
SD^a^			0.39	0.61	0.34	0.54	0.03	0.19	0.07	− 0.14

The slope is the linear regression coefficient indicating selection gain per cycle for DH, S_0_, or S_1_ populations within replications R1 and R2. N refers to the number of genotypes per population

^*^Significant at the 0.05 probability level

^a^Slope for C1-DH—C3-DH expressed as standard deviation of C0

We used linear regression analyses to evaluate selection response. The regression was performed on the following sets: (i) GEBVs of the heterozygous genotypes from C1 to C2, (ii) GEBVs of the DH genotypes from C1 to C3, and (iii) adjusted entry means of DH lines from C1 to C3. The linear model was

4 $$y_{ijr} = \beta_{r} + \gamma_{r} c_{j\left( r \right)} + e_{ijr}$$

where ${y}_{ijr}$ is the GEBV or adjusted entry mean of genotype *i* from cycle *j* in replication *r,* with *r* = 1 for R1 and *r* = 2 for R2. ${\beta }_{r}$ refers to the intercept, ${\gamma }_{r}$ to the selection response per cycle in replication *r* and ${c}_{j(r)}$ is the predictor variable indicating cycle nested within replication *r*. ${e}_{ijr}$ is the residual error with ${e}_{ijr} \boldsymbol{ }\sim \text{ iid }N(0,{\sigma }_{e}^{2})$. In addition to testing H_0_: ${\gamma }_{r}>0$, we also tested H_0_: ${\gamma }_{1}= {\gamma }_{2}$ by a likelihood ratio test comparing models with and without nesting of the cycle effect in replication *r*.

In addition, we tested differences among phenotypic means ${\mu }_{p}$ (*p* = 1–9, Eq. 3) of the DH populations with a global Wald test. If significant, we performed pairwise t tests between the means of the populations within each replication taking heterogeneous variances and degrees of freedom into account as described by Snedecor and Cochran (1989). Response to selection is expressed in units of the genetic standard deviation of C0r. Heterogeneity of genetic variance components ${\sigma }_{g(p)}^{2}$(Eq. 3) between pairs of populations was tested with a likelihood ratio test. Pearson correlations among traits were calculated for each population. To account for multiple testing, we applied the Bonferroni–Holm correction (Holm 1979).

### Prediction and retraining

Prediction abilities were determined for populations C0r and C1-DH to C3-DH as correlations between the GEBVs and adjusted entry means across environments in each population. For comparison among traits, we calculated prediction accuracies (*ρ*) by dividing prediction abilities by the square root of the trait heritability calculated across populations (Dekkers 2007). To evaluate factors influencing prediction accuracy, we calculated *ρ* when training the prediction model on the full data set PKL and on reduced or extended training data sets. In the reduced training sets, we excluded dataset C0r (PKL\C0r) and the selected founder lines (PKL\(C0r, C0sel)) (Table 2). For analyzing the effect of retraining, we extended training sets by successively adding data sets C1-DH and C2-DH (Table 3).

**Table 2:** Prediction accuracies in populations C0r and C1-DH to C3-DH

Training set	N_TS_	Prediction set	PH_V4		PH_V6		PH		FF	
			R1	R2	R1	R2	R1	R2	R1	R2
PKL	899	C0r	0.52		0.61		0.76		0.80	
PKL\C0r	794		0.33		0.41		0.59		0.57	
PKL\(C0r, C0sel)	777		0.33		0.40		0.59		0.57	
PKL\C0r	794	C1-DH	0.37	0.20	0.45	0.28	0.52	0.56	0.56	0.61
PKL\(C0r, C0sel)	777		0.18	0.19	0.31	0.25	0.40	0.49	0.46	0.53
PKL\C0r	794	C2-DH	0.26	− 0.05	0.24	− 0.07	0.52	0.47	0.60	0.56
PKL\(C0r, C0sel)	777		0.18	− 0.10	0.19	− 0.09	0.49	0.47	0.57	0.50
PKL\C0r	794	C3-DH	0.24	0.37	0.28	0.39	0.43	0.56	0.56	0.50
PKL\(C0r, C0sel)	777		0.19	0.29	0.26	0.34	0.42	0.56	0.55	0.48

Training sets were the DH population derived from three landraces (PKL), PKL excluding data set C0r (PKL\C0r), and PKL excluding C0r and selected founder lines C0sel (PKL\(C0r, C0sel)). Traits are plant height in the V4, V6, and final growth stages (PH_V4, PH_V6, PH) and days to female flowering (FF). The training sets were phenotyped in 2017/18. The prediction sets were phenotyped in 2022/23. N_TS_ refers to the number of DH lines in the training set

**Table 3:** Prediction accuracies in populations C1-DH to C3-DH with retraining

Training set	Prediction set	N_TS_		PH_V4		PH_V6		PH		FF	
		R1	R2	R1	R2	R1	R2	R1	R2	R1	R2
PKL\C0r	C1-DH	794	794	0.37	0.20	0.45	0.28	0.52	0.56	0.56	0.61
PKL\C0r	C2-DH	794	794	0.26	− 0.05	0.24	− 0.07	0.52	0.47	0.60	0.56
PKL\C0r + C1		894	894	0.36	0.14	0.39	0.09	0.57	0.51	0.62	0.61
C1		100	100	0.22	0.18	0.41	0.14	0.53	0.31	0.56	0.43
PKL\C0r	C3-DH	794	794	0.24	0.37	0.28	0.39	0.43	0.56	0.56	0.50
PKL\C0r + C1		894	894	0.28	0.46	0.33	0.47	0.41	0.58	0.58	0.57
PKL\C0r + C2		898	905	0.23	0.37	0.32	0.34	0.51	0.60	0.62	0.58
PKL\C0r + C1C2		998	1005	0.26	0.44	0.36	0.43	0.48	0.66	0.65	0.61
C1		100	100	0.21	0.35	0.26	0.42	0.30	0.37	0.48	0.45
C2		104	111	0.11	0.29	0.16	0.14	0.36	0.43	0.54	0.46
C1C2		204	211	0.20	0.40	0.27	0.41	0.38	0.55	0.65	0.54

The training set was the DH population derived from three landraces excluding C0r (PKL\C0r). For prediction sets C2 and C3, scenarios of successive retraining with the DH populations of the previous cycle are shown (+ C1, + C2, + C1C2). Traits are plant height at the V4, V6, and final growth stages (PH_V4, PH_V6, PH) and days to female flowering (FF). PKL\C0r was phenotyped in 2017/18. The C1-DH to C3-DH populations were phenotyped in 2022/23. For the retraining sets, adjusted entry means from 2017/18 and 2022/23 were combined and adjusted via common checks. N_TS_ refers to the number of DH lines in the training set

Since the phenotypic data of the training and prediction sets were assessed in different environments, we evaluated the effect of genotype × environment interaction on *ρ*. This was possible because data set C0r was evaluated in the years 2017/18 and 2022/23. We trained the model on data set PKL\C0r phenotyped in 2017/18 and compared prediction accuracies obtained from C0r phenotypes from 2017/18 with those from 2022/23.

The statistical model for predicting the GEBVs in the initial selection step was trained on a 600 k SNP chip, while genomic selection in subsequent cycles (C1-S_1_ and C2-S_0_) relied on an 11 k chip. To assess the potential effect of marker density on prediction accuracy, we calculated Pearson correlation coefficients for the GEBVs obtained with the two approaches in the training set (PKL, N = 899). Additionally, we compared GEBVs from training on all DH lines (PKL, N = 899) versus those from training on DH lines from landrace PE only (C0, N = 402), using both 600 k and 11 k SNPs. For plant height in V4, V6, and final growth stages, we also correlated the GEBVs obtained from a multi-trait model with those from a single-trait model for the selection traits (600 k and 11 k, PKL, N = 899 and C0, N = 402).

To assess if differences in prediction ability between pairs of prediction sets were significant, we used a bootstrap analysis (Efron 1979). To this end, sampling with replacement was performed in pairs of prediction sets, calculating the difference between their prediction abilities in two scenarios: (i) in samples of prediction set C0r, prediction abilities from 2017/18 and 2022/23 were paired; (ii) for all possible pairs of C1-DH to C3-DH prediction sets, independent samples from each population were paired. Differences in prediction ability between prediction sets were considered significant if zero was not included in the interval spanned by the 2.5% and 97.5% quantiles of the bootstrap distribution.

## Results

### Model training for selection

Pearson correlation coefficients between GEBVs based on model training on all DH lines (PKL, N = 899) and those using phenotypic data from PE only (C0, N = 402) exceeded 0.98 for all traits. Similarly, GEBVs obtained with the 600 k and the 11 k chip were highly correlated (*r* > 0.98) for all traits. Correlations between GEBVs calculated using the multi-trait model and those from the single-trait model exceeded 0.99 for the three selection traits.

### Molecular differentiation of cycles C0 to C3

**Fig. 2:**
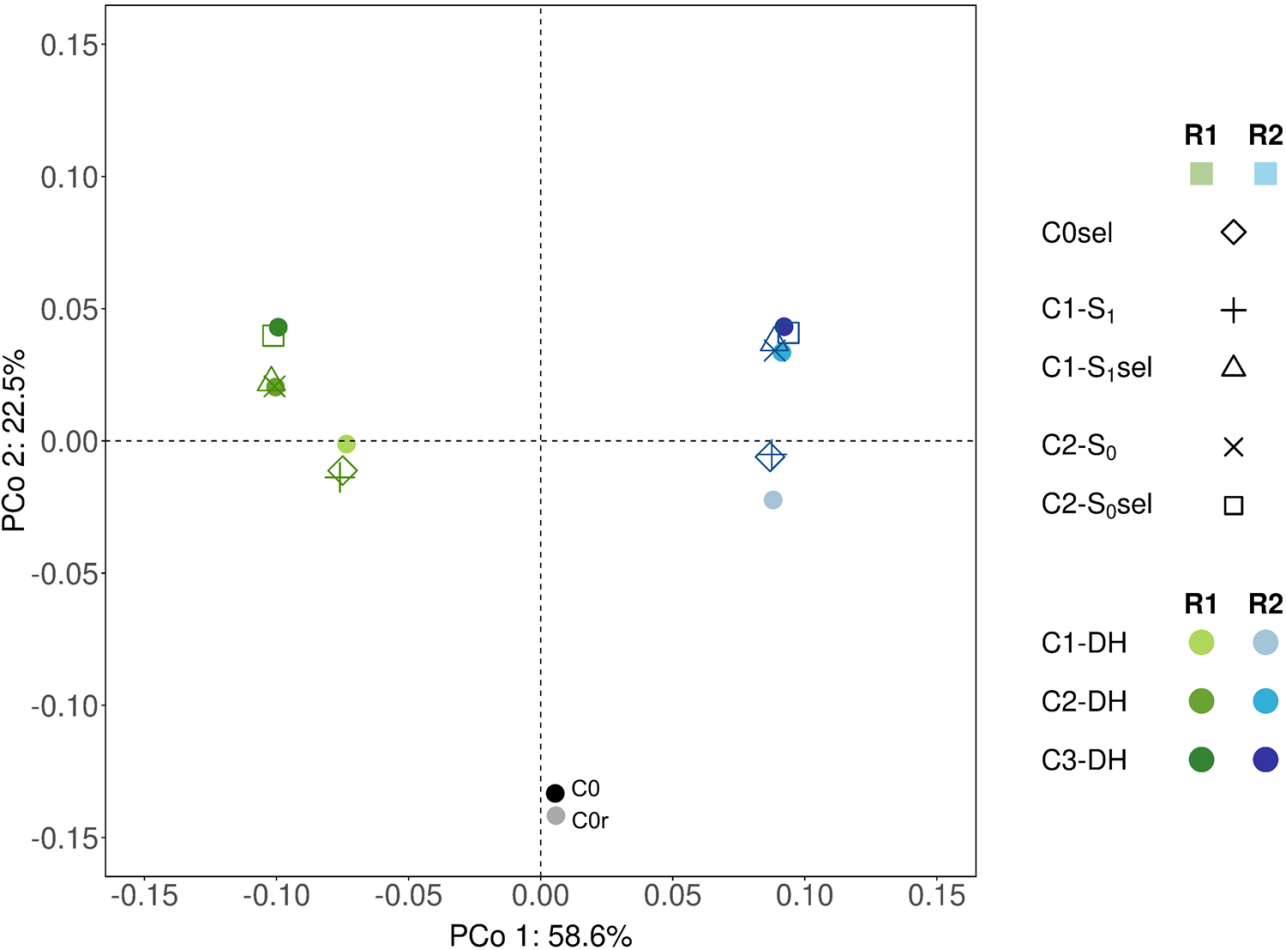
Principal coordinate analysis (PCoA) of all populations from the replicated selection experiment based on modified Rogers’ distances calculated from allele frequencies of the populations. C0 and C0r refer to the base population and a subset of 105 DH lines randomly sampled from it; C1-S_1_ and C2-S_0_ are the populations comprising the selection units; C0sel, C1-S_1_sel, and C2-S_0_sel, the selected candidates per cycle; C1-DH, C2-DH, and C3-DH populations were derived after recombination of the selected candidates of the previous cycle

The principal coordinate analysis based on MRD values between populations separated the two replications with the first two coordinates explaining 58.6% and 22.5% of the total molecular variance, respectively (Fig. 2). In both replications, C0 and C0r were clearly separated from all other cycles. In R1, cycles C1 to C3 were separated by both coordinates, while in R2, C1 to C3 differed mainly in PC2. Populations C1-DH and C1-S_1_ clustered closely together in R1 and R2, indicating that on the whole-genome level C1-DH represented C1-S_1_ very well. The same was true for C2-DH that could not be separated from C2-S_0_. As expected, the founder lines selected in C0 clustered with C1-DH and the individuals selected in C1-S_1_ and C2-S_0_ with C2-DH and C3-DH, respectively.

### GEBVs of selection units and DH lines from cycles C0 to C3

Means of GEBVs presented in Table 1 were similar for selection units (C1-S_1_, C2-S_0_) and DH lines (C1-DH, C2-DH) extracted from them, corroborating results from molecular analyses that DH lines are a representative sample of the selection units. For early plant height in growth stages V4 and V6, GEBVs increased continuously from C0 to C3. The largest increase was observed from C0 to C1 amounting to 1.5–1.9 SD for the two traits and replications. When fitting a linear regression model with cycle as predictor variable (C1 to C3), regression coefficients indicated a significant increase in GEBVs from C1-DH to C3-DH in both replications (0.3–0.6 SD per cycle, Table 1). For final plant height and flowering time, the regression coefficient in R1 was not significant but a significant change in GEBVs was observed in R2. Selection gains were diminishing from cycles C0 to C3 as would be expected for genomic selection without retraining.

### Adjusted entry means of DH lines from cycles C0 to C3

DH lines representing cycles C1 to C3 were evaluated in multi-environment field trials in years 2022 and 2023 together with the 105 DH lines of C0r to quantify realized selection gain for the traits under selection. To serve as benchmark, C0r needs to be a representative subsample of C0. In 2017/18, mean and variance estimates for the selection traits did not differ significantly between C0r and C0 (Table S3), indicating that C0r can serve as a reference for evaluating selection gain from C0 to C3. Molecular analyses corroborated these findings with C0 and C0r being close together on the first and second principal coordinates (Fig. 2).

**Fig. 3:**
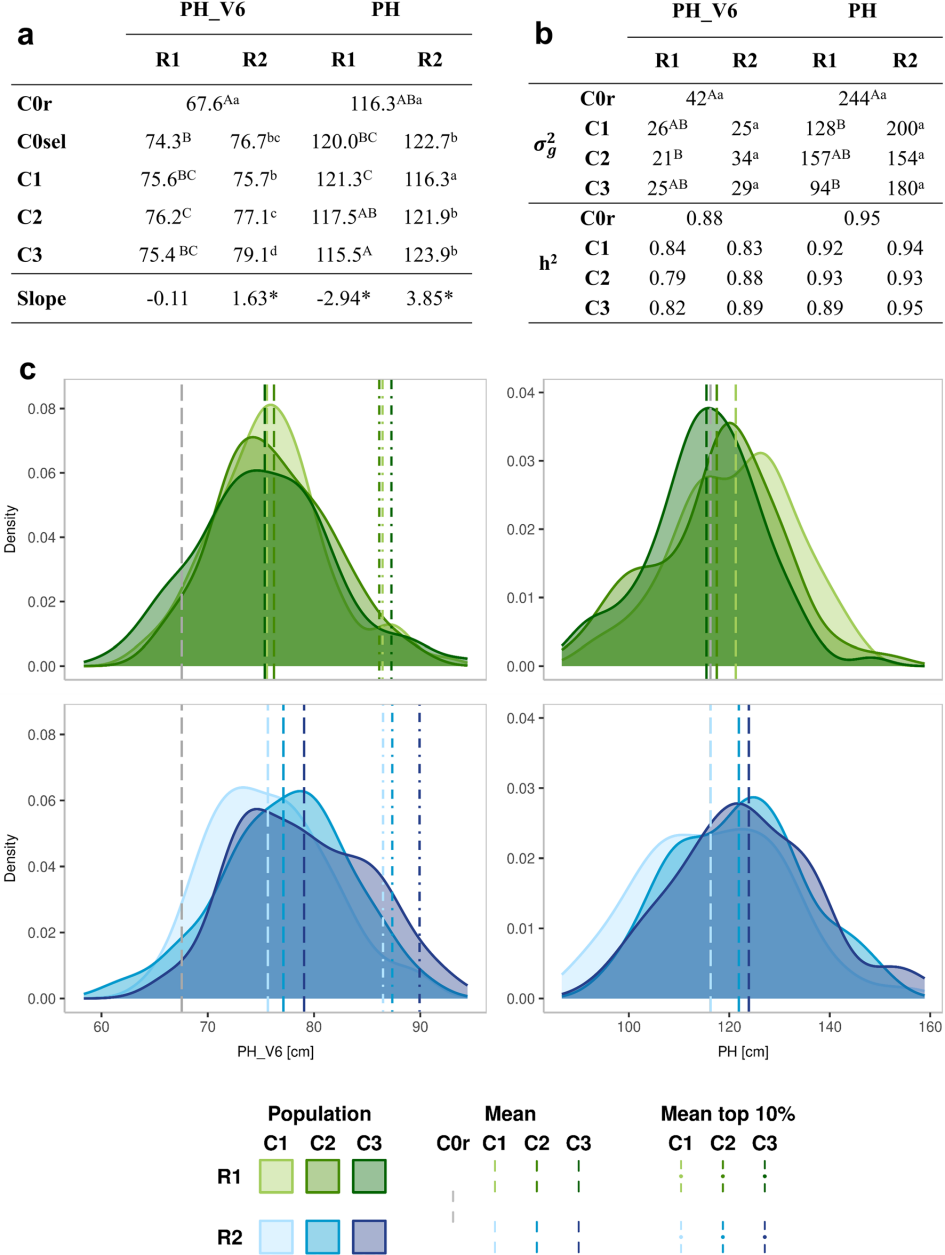
Response to selection based on adjusted entry means of populations in two replications (R1 and R2) of the experiment.** a** Means of DH populations for plant height in V6 and final growth stages (PH_V6, PH) and the coefficient of linear regression indicating selection response per cycle from C1 to C3 (Slope). **b** Genetic variances and heritabilities. Different letters (upper case for R1; lower case for R2) in **a** and **b** indicate significant differences between populations (*P* < *0.05*). **c** Density distribution of adjusted entry means of DH lines in each population. The dashed lines indicate the population means, the dashed-dotted lines the means of the best performing 10% per population for PH_V6

Selection gains predicted from GEBVs were only partially corroborated by realized gains estimated from adjusted entry means. A large increase was observed from C0 to C1 for early plant height in growth stages V4 and V6 (1.2–1.4 SD; corresponding to 14% of the mean in C0 for PH_V4 and 12% for PH_V6) (Fig. 3, Fig. S2). Results from linear regression with cycle as predictor variable (C1 to C3) in R2 revealed a significant positive increase of all three selection traits (0.20 SD or 2.1% per cycle for PH_V4; 0.25 SD or 2.4% for PH_V6; 0.25 SD or 3.3% per cycle for PH). In R1, no significant change of adjusted entry means was observed for early plant height from C1 to C3 and for final plant height a significant decrease was observed. The more pronounced increase of population means for early plant height V6 in R2 compared to R1 was also reflected in a higher mean of the best performing 10% DH lines especially in C3 (Fig. 3).

Genetic variances of the traits under selection decreased, but differences were not significant across cycles, except for final plant height in R1 (Fig. 3). Trait correlations remained stable across cycles (Table S4). Early plant height in stages V4 and V6 were highly correlated, while their mutual correlations with final plant height were intermediate to low. Correlations with flowering time were not significant with any of the traits under selection.

### Prediction accuracies in DH populations of cycles C0 to C3

Prediction accuracies of the four traits under study obtained in DH populations from all cycles are presented in Table 2. Bootstrap analyses comparing prediction abilities between cycles yielded large 95% confidence intervals, ranging from 0.38 (PH and FF) to 0.58 units (PH_V4) (Table S5).

For a fair comparison of accuracies in prediction sets C1 to C3 with C0r, we excluded the 105 DH lines of C0r from PKL and trained the prediction model on the 794 remaining lines (PKL\C0r). For prediction set C0r, accuracies decreased consistently for all traits by about 0.2 units compared to training with PKL. Prediction sets C1 to C3 were affected to a smaller extent (data not shown). Excluding also the DH founder lines selected in C0 (PKL\(C0r, C0sel)) reduced accuracies further, particularly in C1 and R1.

In the comparisons across cycles, prediction accuracies for final plant height and flowering time with training set PKL\C0r decreased only slightly from C0r to C3 in both replications. The accuracies for the selected traits early plant height in growth stages V4 and V6 varied considerably more across cycles. To avoid redundancy, we focus on early plant height in growth stage V6. In R1 with training set PKL\C0r, accuracies remained stable from C0r (*ρ* = 0.41) to C1 (*ρ* = 0.45) but decreased in C2 and C3 (*ρ* = 0.24, 0.28). Based on the 95% bootstrap confidence intervals, differences between C0r and C2 and C3 were not significant. In R2, accuracies decreased to *ρ* = 0.28 in C1, dropped to nearly zero in C2, and increased again in C3 to values higher than in C1 (*ρ* = 0.39). All comparisons involving C2 in R2 were significant in the bootstrap analysis (Table S5).

To assess the impact of genotype × environment interactions on prediction accuracy, we trained the model on PKL\C0r and predicted the GEBVs of the 105 DH lines from C0r. Prediction accuracies were slightly higher (maximum of 0.08) with C0r phenotypic data from 2017/18 than with phenotypic data from 2022/23, but bootstrap analysis yielded no significant differences (data not shown).

For prediction sets C2 and C3, we evaluated the effect of retraining the prediction model with DH lines from previous cycles (Table 3**)**. In prediction set C2, accuracies increased when DH lines derived from cycle C1 were added to the training set (PKL\C0r + C1). In prediction set C3, this was also true for retraining with C1, but retraining with C2 decreased accuracies in some cases (e.g., early plant height in growth stage V6 in R2).

## Discussion

Rapid recurrent genomic selection has been studied in simulations and empirical studies of breeding populations (Dreisigacker et al. 2023; Gorjanc et al. 2018), but inferences from breeding populations cannot be directly applied to pre-breeding of landraces. While breeding populations are more or less closed populations with a high degree of relatedness, landraces were propagated by panmixia and mild selection of farmers over long periods of time. Breeding populations are genetically optimized while landraces are expected to harbor deleterious alleles, possibly in high linkage disequilibrium (LD) with favorable alleles. In landraces, the focus lies on short-term population improvement by rapidly increasing the frequency of positive alleles for the target traits and closing the performance gap to breeding populations.

Genomic selection has been suggested as an efficient method to close this performance gap (Allier et al. 2020; Gorjanc et al. 2016; Sanchez et al. 2023). However, in silico studies make strong assumptions about the genetic architecture of the trait under selection. These include the number of QTL, their distribution across the genome, allele frequencies at the QTL, the distribution of allele effect size, as well as allelic interactions with each other and the environment. In studies on genome-based backcrossing, Bernardo (2009, 2016) demonstrated that the outcome of simulation scenarios differed fundamentally depending on the simulated trait architecture. In contrast, empirical selection experiments provide insights that are grounded in real-world scenarios, but they are highly resource consuming and the effect of selection is difficult to distinguish from random drift in un-replicated experiments. Nevertheless, for a specific breeding scheme they can give first insights on the realized response to selection, changes in genetic variances and prediction accuracies across selection cycles by comparing selected and unselected traits.

Here, we performed a replicated genomic selection experiment in a maize landrace. The goal was to investigate for polygenic traits how much selection gain could be realized in three cycles of selection and recombination without retraining the prediction model. We chose early plant development as a model trait because it is a major sustainability factor in maize production, can be evaluated in large field trials on a line per se basis, and has high heritability. In previous studies, we could also show that based on SNPs or haplotypes intermediate prediction accuracies can be obtained for quantitative traits in these landrace populations (Hölker et al. 2022, 2019; Lin et al. 2024).

### Selection response decreases rapidly across cycles

We evaluated the selection response for two traits under directional selection, and one trait under stabilizing selection over three cycles of selection and recombination. Directional selection led to a large response for early plant height in growth stages V4 and V6 from C0 to C3 with only a small correlated response in final plant height and flowering time. The choice of the training set (three landraces versus PE only), the marker density (600 k versus 11 k), and the statistical model (multi- versus single-trait) had no or only a marginal effect on the choice of DH lines (C0sel) that were selected to initiate the selection process. We conclude that multi-trait selection in landrace populations can be highly successful if the founder parents for the selection cycles are chosen based on a large number of landrace-derived DH lines with high density genotypic data and multi-environment phenotypes.

The largest response was seen for selection in the base population (C0) in which GEBVs largely reflected the phenotypic values of the selection candidates due to high trait heritabilities and limited relatedness of DH lines derived from a panmictic landrace. In the succeeding two selection steps without phenotypes, the observed response per cycle was reduced (Fig. 3). This result was also reflected at the molecular level by the limited differentiation of the populations in the more advanced cycles (Fig. 2). There are several explanations for these outcomes. In pre-breeding of landraces, the goal is short-term, i.e., a few best genotypes are needed for introducing novel alleles for target traits into elite germplasm. Thus, we followed results from simulation studies that suggest to select and recombine only few progenies in each cycle if the primary goal is to increase the population mean (Bernardo 2010). Due to the high selection intensity applied in the base population in both replications of the experiment, it is likely that positive alleles at QTL with large effects or intermediate allele frequencies were fixed in the selected DH lines (C0sel). Consequently, in the following cycles selection operated mainly on QTL with smaller effects and more extreme allele frequencies predicted with lower precision in training set PKL. In cycle C1, the selection candidates (S_1_ plants) with the best GEBVs originated from only few of the 45 possible families corroborating this assumption. We therefore restricted the number of progenies per C0sel founder line to 15 when selecting the S_1_ plants in cycle C1. As is known from optimum contribution selection (Woolliams et al. 2015), introducing a side condition to control relatedness reduces selection gain and could be an additional explanation for the smaller increase from C1 to C3 as compared to the first round of selection. Another side condition in our selection scheme was that the increase in early plant height should not lead to a correlated response in final plant height. We applied a multi-trait selection criterion (Eq. 2) that most likely diminished the selection pressure on early plant height compared to an alternative scenario of directional truncation selection on early plant height in growth stages V4 and V6 without stabilizing selection for final plant height. A regression analysis with the selection criterion as response variable and its three components entering as predictor variables showed that PH_trans accounted for about 20 to 30% of the variance of the selection criterion in each selection step (Table S6).

A further explanation for the reduced genetic gain in C1 and C2 could be the differences in genetic structure between the training set PKL and the selected populations. Habier et al. (2013) showed that prediction accuracy is a function of three different sources: ancestral linkage disequilibrium (LD), pedigree relatedness between the training and the prediction set, and co-segregation between markers and QTL due to linkage. In our study, the model was trained on DH lines derived from S_0_ plants of a random mating population PE (C0). Thus, the GEBVs of C1-S_1_ and C2-S_0_ selection units were predicted based on the ancestral LD of the base population C0 and on their relatedness with the C0sel founder lines. Without retraining the prediction model, co-segregation of markers and QTL cannot be accounted for in prediction of GEBVs in fast-track schemes as the founder lines do not inform about linkage between markers and QTL in C1 to C3 (Habier et al. 2013). We expect therefore a decreasing selection response per cycle as pedigree relatedness between founder lines and selection units is decreasing. This was reflected in GEBVs (Table 1) but much more so in the adjusted means (Fig. 3). We hypothesize that LD between markers and QTL not present in C0 but emerging in C1-S_1_ and C2-S_0_ selection units as a result of strong selection and controlled mating is the reason for this discrepancy. This hypothesis is corroborated by the prediction accuracies estimated from DH lines in cycles C0 to C3 discussed in the following section.

### Prediction accuracies decrease strongly for traits under directional selection

Prediction abilities or their corresponding accuracies have been shown to be associated with high statistical uncertainty (Gianola and Schön 2016) which was also found in this study (Table S5). Therefore, the differences in accuracies found in this study need to be interpreted with caution, but a few results stand out. These are the differences in accuracies for the traits under directional selection and final plant height and flowering time, the high volatility of accuracies in R2, and the differences between replications.

For flowering time and final plant height, changes in prediction accuracies across cycles were negligible (Table 2), indicating that for these traits the decrease in relatedness from C0 to C3 had no or only a small effect and that accuracies were driven mainly by ancestral LD. In contrast, directional selection had a strong effect on prediction accuracies as can be seen especially in C2 of R2 for early plant height (V4 and V6). If we assume the genetic architecture of all investigated traits to be highly polygenic, the effects of ancestral LD and relatedness should be similar across traits. Thus, we hypothesize that the sharp drop in prediction accuracy from C0 to C2 for the traits under directional selection is the result of LD between markers and QTL induced by selection and not represented in the DH lines of the training set (PKL).

Further evidence for the validity of this hypothesis comes from retraining the prediction model based on phenotypes from C1-DH and C2-DH for predicting DH lines in C3. In our study, retraining was expected to lead to an increase in prediction accuracies for several reasons. Firstly, part of the training set is now evaluated in the same environment as the prediction set (2022/23), thus attenuating the effects of genotype × year interactions. Secondly, DH lines from C1 and C2 represent the selection units of the previous cycles and are expected to be more closely related to those in C3 than the DH lines from PKL. Thirdly, the DH lines from C1 and C2 should reflect the sampling LD of the founder lines C0sel to a certain extent. When updating the prediction model with DH lines from C1, accuracies in prediction set C3 increased, but they decreased in R2 when DH lines from C2 were included in the training set. It was also notable that accuracies in prediction set C3 of R2 were almost as high as those in prediction set C0r and increased even more when retraining with C1-DH lines pointing to similar co-segregation of markers and QTL in C3-DH and C1-DH lines. This is corroborated by training the model on C1-DH lines only for predicting C3. Despite the small sample size of N = 100, accuracies were almost the same as for training with PKL\C0r with N = 794.

Given that several factors influence prediction accuracies in rapid cycling prediction, insights on their respective contributions warrants further research. Ancestral LD, relatedness or genotype × environment interactions should affect the two replications in a similar way. Here, we observed pronounced differences in selection response and prediction accuracies between R1 and R2, but mainly for the traits under directional selection. These differences between replications must therefore mainly be attributed to the sampling of different C0sel lines for initiating R1 and R2.

### Genetic variance and diversity decreased moderately across cycles

We hypothesized that the high selection intensities in all three cycles had a major impact on prediction accuracies and on the selection response. Under intense selection, a decrease in genetic variance is expected for the traits under selection. In both replications, genetic variance of the traits under selection decreased, but differences were significant only for final plant height in R1 under stabilizing selection. This rather moderate decrease of genetic variance could be the result of a compensation effect. It is possible that genetic variance was increased for the selection traits in the more advanced populations by changes in allele frequencies toward intermediate values or by release of new additive genetic variance by epistatic interactions in a more constrained genetic space, as has been suggested for breeding populations with small effective population size (Technow et al. 2021).

We also observed that the average genomic relationship of selection units and DH lines increased over cycles (data not shown). To manage genetic diversity in recurrent selection, optimal contribution or optimal cross selection has been proposed (Allier et al. 2019; Gorjanc et al. 2018; Woolliams et al. 2015). When selecting the C0sel founder lines, controlling for coancestry was not applicable as they were all derived from a random mating population and DH lines were assumed to be unrelated. In the second and third selection steps, we put an upper bound on the number of progenies per C0sel founder line, but managing diversity under selection is still an open question and in landraces even more so than in breeding populations. As Gorjanc et al. (2018) pointed out, it is still unclear *“which genetic diversity should be preserved and which discarded”* as measuring diversity on a whole-genome basis does not necessarily reflect the diversity at QTL for the traits under selection.

## Conclusions

Our results suggest that rapid recurrent genomic selection can be highly successful if a large population with high-quality genotypic and phenotypic data is available for model training. However, without retraining, the selection response in advanced cycles will be highly variable due to parental sampling and the resulting genetic drift. Retraining the prediction model as soon as possible is advisable requiring the combination of high throughput genotyping, speed breeding, and the DH technology. To devise optimal retraining strategies for rapid cycling recurrent selection, more in-depth analyses of the genomic regions targeted by selection, the role of the mating design, and the generation of LD in highly selected populations are required. These investigations are beyond the scope of this study and will be addressed elsewhere.

## Supplementary Information

Below is the link to the electronic supplementary material.

Supplementary file1 (PDF 528 KB)

## Data Availability

All data and material are available through material transfer agreements upon request.
