# Supplementary material for: Rapid cycling genomic selection in maize landraces
Source: Theor Appl Genet. 2025 Mar 17;138(4):75. doi: 10.1007/s00122-025-04855-6 (PMC11913984; doi:10.1007/s00122-025-04855-6)
Supplement: Supplementary file 1 — Supplementary file1 (PDF 528 KB) [file 122_2025_4855_MOESM1_ESM.pdf]

Theoretical and Applied Genetics

Rapid cycling genomic selection in maize landraces

Clara Polzer[1], Hans-Jürgen Auinger[1], Michelle Terán Pineda[1], Armin C. Hölker[1,a], Manfred Mayer[1,b], Thomas Presterl[2], Carolina Rivera Poulsen[1], Sofia da Silva[2], Milena Ouzunova[2], Albrecht E. Melchinger[1,3], Chris-Carolin Schön[*1]

[1] Plant Breeding, TUM School of Life Sciences, Technical University of Munich, Freising, Germany, 85354

[2] KWS SAAT SE & Co. KGaA, Einbeck, Germany, 37574

[3] Institute of Plant Breeding, Seed Science and Population Genetics, University of Hohenheim, Stuttgart, Germany, 70593

[a] present address: KWS SAAT SE & Co. KGaA, 37574 Einbeck, Germany

[b] present address: Bayer Crop Science, 46325 Borken, Germany

[*]Corresponding author

Email address: chris.schoen@tum.de

Supplemental tables

Table S1 Description of data sets used for model training, selection, and evaluation of selection response. Given are the sample size (N), the number of parents (N parents), the number of families (N families), the average number of progenies per family (N progenies) with minimum and maximum per population in brackets and the years for genotypic or phenotypic evaluation of each data set. The training set is derived from three open-pollinated landraces (OPV).

Data set	N	Origin/ N parents	N families	N progenies	Years of evaluation
**Training set (DH lines)**					2017/2018
PKL	899	Landraces Petkuser Ferdinand Rot (PE), Kemater Landmais Gelb (KE), Lalin (LL)		OPV derived	
C0	402	Landrace PE		OPV derived	
**Selection**					2019/2020
R1					
C1-S_1	1003	10	44	22.80 (11, 26)	
C2-S_0	1005	44	22	45.68 (6, 69)	
R2					
C1-S_1	1001	8	25	40.04 (19, 42)	
C2-S_0	906	36	18	50.33 (3, 154)	
**DH lines**					2022/2023
C0r	105	Landrace PE		OPV derived	
R1					
C1-DH	100	10	26	3.84 (1, 10)	
C2-DH	104	36	18	5.78 (1, 27)	
C3-DH	90	28	14	6.43 (1, 14)	
R2					
C1-DH	100	8	20	5.00 (1, 29)	
C2-DH	111	34	17	6.53 (1, 17)	
C3-DH	78	32	16	4.88 (1, 20)	

Table S2 Traits, abbreviations, description of trait collection and environments for each trait.

Trait	Abbreviation	Description	Environments	Number of Environments
Early plant height	PH_V4 and PH_V6	In cm, from ground to longest, manually vertically stretched leaf, in V4 and V6 stage. Averaged over three plants.	ROG 2022<br>EIN 2022/23<br>BBG 2022/23<br>OLI 2022/23	7
Final plant height	PH	In cm, from ground to first branch of the tassel. Averaged over three plants.	ROG 2022<br>EIN 2022/23<br>BBG 2022/23<br>OLI 2022/23	7
Days to female flowering	FF	Days after sowing when 50% of the plants have a fully developed female flower (upper 1-2 cm of the silk visible).	ROG 2022<br>EIN 2022/23<br>BBG 2022/23<br>OLI 2022/23	7

Table S3 Means, genotype (σ_g^2), genotype × environment (σ_{gl}^2) and error (σ_e^2) variance components, each with their standard error, and heritabilities with confidence intervals for data sets C0 and its random subset C0r evaluated in 11 environments in 2017/18 for the selection traits plant height in V4, V6 and final growth stage (PH_V4, PH_V6, PH) and for days to female flowering (FF).

	**PH_V4**		**PH_V6**	
	**C0**	**C0r**	**C0**	**C0r**
**Mean ± SE**	43.8 ± 0.3	43.9 ± 0.5	83.7 ± 0.5	83.8 ± 0.8
$\boldsymbol{\sigma_g^2 \pm}$ **SE**	24.8 ± 2.0	21.5 ± 3.3	77.2 ± 6.0	62.1 ± 9.6
$\boldsymbol{\sigma_{gl}^2 \pm}$ **SE**	18.4 ± 0.7	14 ± 1.1	38.7 ± 1.7	33.4 ± 2.9
$\mathbf{h^2}$(**CI**)	0.91 (0.90, 0.92)	0.92 (0.89, 0.94)	0.93 (0.92, 0.94)	0.92 (0.90, 0.94)

	**PH**		**FF**	
	**C0**	**C0r**	**C0**	**C0r**
**Mean ± SE**	125 ± 1	127 ± 2	79.2 ± 0.2	78.3 ± 0.3
$\boldsymbol{\sigma_g^2 \pm}$ **SE**	285 ± 21	221 ± 33	14.8 ± 1.1	10.2 ± 1.6
$\boldsymbol{\sigma_{gl}^2 \pm}$ **SE**	91 ± 4	95 ± 8	4.2 ± 0.2	3.7 ± 0.4
$\mathbf{h^2}$(**CI**)	0.96 (0.95, 0.96)	0.94 (0.92, 0.96)	0.95 (0.95, 0.96)	0.94 (0.92, 0.95)

Table S4 Phenotypic correlations between the selection traits calculated in DH populations C0r to C3. The selection criterion applied in each cycle aimed at directional selection for early plant height in growth stage V4 and V6 (PH_V4, PH_V6) and stabilizing selection for final plant height (PH).

	**PH_V4 - PH_V6**		**PH_V4 - PH**		**PH_V6 - PH**	
	**R1**	**R2**	**R1**	**R2**	**R1**	**R2**
**C0r**	0.87*		0.43*		0.52*	
**C1**	0.90*	0.88*	0.35*	0.34*	0.42*	0.33*
**C2**	0.82*	0.90*	0.27	0.40*	0.49*	0.49*
**C3**	0.80*	0.76*	0.24	0.26	0.47*	0.40*

* Significant at the 0.05 probability level

Table S5 Bootstrap results for differences in prediction ability between pairs of DH populations obtained with the training set PKL\C0r in replication R1 and R2 (Rep). Q2.5 and Q97.5 are the 2.5% and 97.5% quantiles of the bootstrap distribution. Population pairs with a significant difference in prediction ability (95% bootstrap interval), are marked in bold.

Rep	Population pair	PH_V4			PH_V6			PH			FF		
		**Q2.5**	**Mean**	**Q97.5**	**Q2.5**	**Mean**	**Q97.5**	**Q2.5**	**Mean**	**Q97.5**	**Q2.5**	**Mean**	**Q97.5**
**R1**	**C0r vs. C1**	-0.28	-0.03	0.22	-0.27	-0.03	0.20	-0.12	0.07	0.27	-0.19	0.02	0.23
	**C0r vs. C2**	-0.19	0.07	0.32	-0.10	0.16	0.42	-0.13	0.06	0.25	-0.22	-0.02	0.17
	**C0r vs. C3**	-0.17	0.08	0.34	-0.14	0.12	0.39	-0.05	0.16	0.37	-0.19	0.01	0.21
	**C1 vs. C2**	-0.15	0.10	0.34	-0.05	0.20	0.44	-0.22	-0.01	0.19	-0.24	-0.04	0.17
	**C1 vs. C3**	-0.14	0.12	0.37	-0.10	0.16	0.41	-0.14	0.09	0.30	-0.21	0.00	0.20
	**C2 vs. C3**	-0.24	0.02	0.28	-0.32	-0.04	0.23	-0.12	0.10	0.31	-0.16	0.03	0.22
**R2**	**C0r vs. C1**	-0.16	0.12	0.42	-0.16	0.12	0.40	-0.17	0.04	0.24	-0.23	-0.03	0.17
	**C0r vs. C2**	**0.09**	**0.35**	**0.59**	**0.18**	**0.44**	**0.70**	-0.08	0.12	0.31	-0.19	0.01	0.20
	**C0r vs. C3**	-0.28	-0.04	0.20	-0.24	0.02	0.27	-0.16	0.03	0.22	-0.15	0.07	0.29
	**C1 vs. C2**	-0.07	0.23	0.51	**0.03**	**0.32**	**0.60**	-0.14	0.08	0.30	-0.16	0.04	0.24
	**C1 vs. C3**	-0.45	-0.16	0.12	-0.39	-0.10	0.18	-0.22	-0.01	0.20	-0.12	0.10	0.33
	**C2 vs. C3**	**-0.63**	**-0.39**	**-0.14**	**-0.68**	**-0.42**	**-0.16**	-0.29	-0.09	0.11	-0.15	0.06	0.28

Table S6 Adjusted coefficients of determination ($R^2_{adj.}$) for the multiple regression analyses of the selection criterion SC (see Eq. 2) on the underlying components for three populations. C0 denotes the DH population from landrace PE and C1-S_1 and C2-S_0 the populations comprising the selection candidates in cycles C1 and C2. Predictor variables were the genomic estimated breeding values (GEBVs) for PH_V4, PH_V6, and PH_trans as described in Materials and Methods.

	Population				
**Predictor variable(s)**	**C0**	**C1-S_1**		**C2-S_0**	
		**R1**	**R2**	**R1**	**R2**
**PH_V4**	0.70	0.65	0.71	0.68	0.50
**PH_V6**	0.74	0.65	0.71	0.66	0.57
**PH**	0.38	0.21	0.43	0.17	0.21
**PH_trans**	0.29	0.20	0.20	0.20	0.31
**PH_V4 + PH_V6**	0.74	0.69	0.73	0.70	0.58
**PH_V4 + PH**	0.71	0.65	0.71	0.68	0.50
**PH_V4 + PH_trans**	0.98	0.95	0.98	0.96	0.95
**PH_V6 + PH**	0.74	0.65	0.72	0.67	0.57
**PH_V6 + PH_trans**	0.98	0.95	0.98	0.96	0.93
**PH_V4 + PH_V6 + PH**	0.74	0.69	0.73	0.70	0.59

Supplemental figures

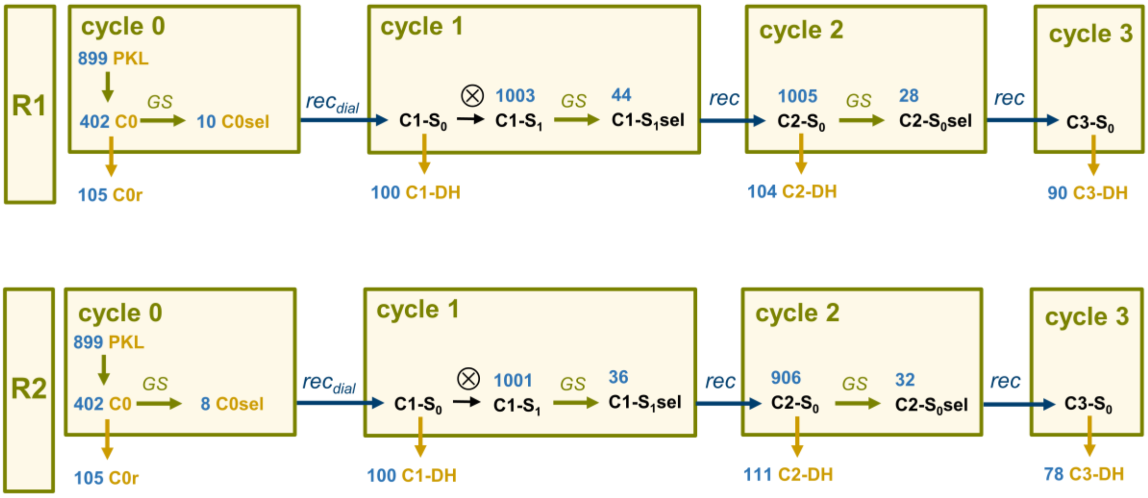

Fig. S1 Rapid cycling genomic selection (GS) scheme for early and final plant height traits, replicated twice (R1, R2). Following model training with a set of 899 DH lines, the best C0sel DH lines from population PE (C0) were selected based on a multi-trait selection criterion, recombined in a diallel crossing scheme and selfed once to produce the C1-S_1 population. Subsequently, two cycles of GS and mating of the selected candidates in pairs were conducted. From each selection cycle, DH lines were sampled (C0r) or produced (C1-DH, C2-DH, C3-DH). Blue digits indicate the number of selection units, selected fractions and phenotyped and genotyped DH lines

a

	PH_V4		FF	
	R1	R2	R1	R2
**C0r**	35.7^{Aa}		80.9^{Aa}	
**C0sel**	40.4^{B}	41.4^{bc}	82.3^{B}	81.4^{a}
**C1**	40.7^{BC}	40.8^{b}	80.9^{A}	81.2^{a}
**C2**	41.0^{BC}	41.2^{b}	81.5^{AB}	81.1^{a}
**C3**	41.6^{C}	42.3^{c}	83.6^{C}	81.1^{a}
**Slope**	0.39	0.76*	1.31*	-0.05

b

		PH_V4		FF	
		R1	R2	R1	R2
	**C0r**	13^{Aa}		17^{Aa}	
σ_g^2	**C1**	10^{A}	10^{a}	16^{A}	22^{a}
	**C2**	9^{A}	13^{a}	18^{A}	13^{a}
	**C3**	10^{A}	11^{a}	21^{A}	20^{a}
	**C0r**	0.86		0.95	
h^2	**C1**	0.82	0.81	0.94	0.95
	**C2**	0.82	0.86	0.94	0.92
	**C3**	0.82	0.82	0.96	0.95

c

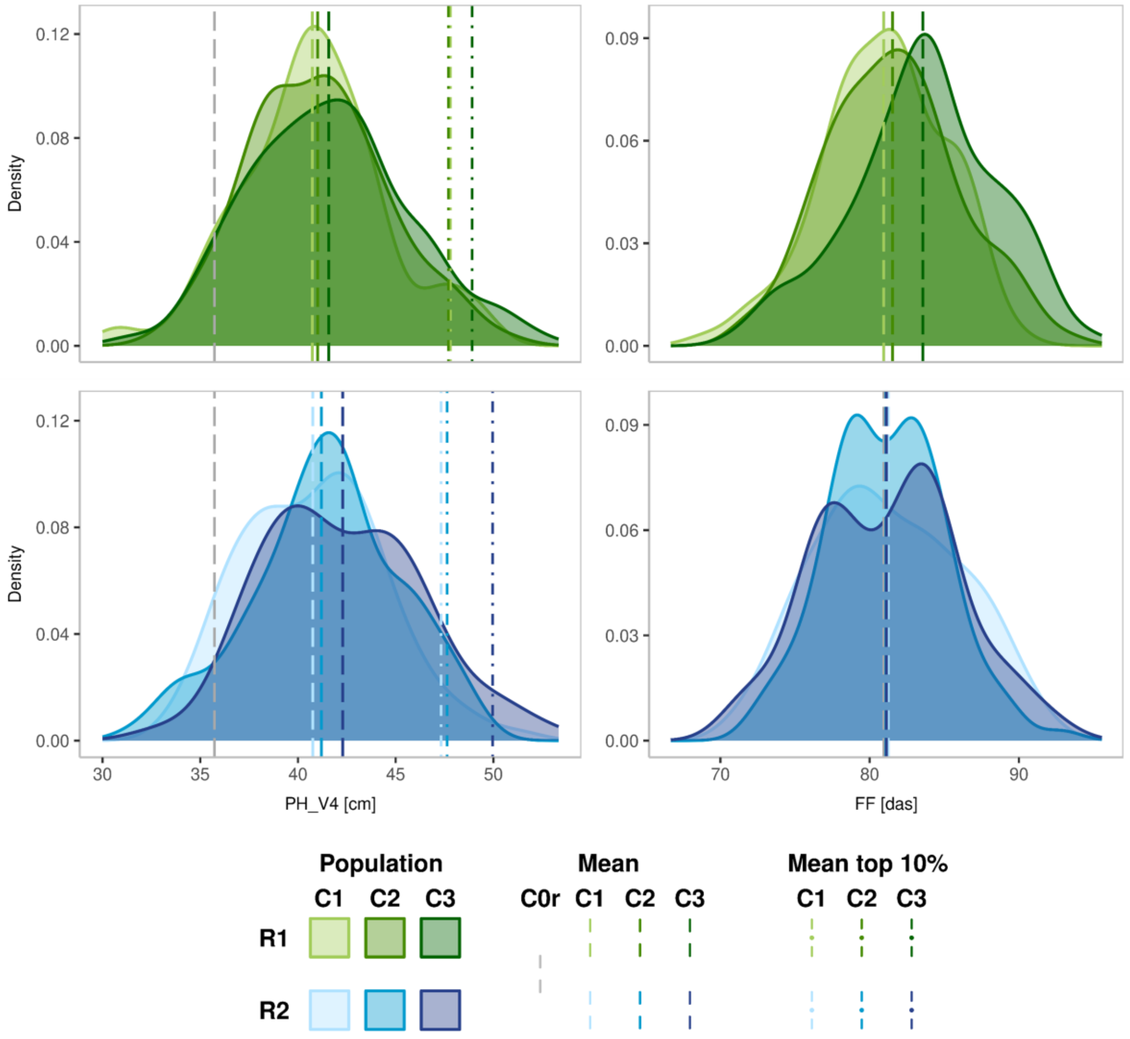

Fig. S2 Response to selection based on adjusted entry means of populations in two replications (R1 and R2) of the experiment. **a** Means of DH populations for plant height in growth stage V4 (PH_V4) and days to female flowering (FF), and the coefficient of linear regression indicating selection response per cycle from C1 to C3 (Slope). **b** Genetic variances and heritabilities. Different letters (upper case for R1; lower case for R2) in **a** and **b** indicate significant differences between populations ($P < 0.05$). **c** Density distribution of adjusted entry means of DH lines in each population. The dashed lines indicate the population means, the dashed-dotted lines the means of the best performing 10% per population for PH_V4
